# Electrocardiographic imaging metrics to predict the risk of arrhythmia in patients with ischemic cardiomyopathy

**DOI:** 10.1002/joa3.70024

**Published:** 2025-02-17

**Authors:** Azizah Puspitasari Ardinal, Holly P. Morgan, Mark Elliott, Martin Bishop, Christopher Aldo Rinaldi, Divaka Perera

**Affiliations:** ^1^ British Heart Foundation Centre of Research Excellence at the School of Cardiovascular and Metabolic Medicine & Sciences King's College London London UK; ^2^ School of Biomedical Engineering and Imaging Sciences King's College London UK; ^3^ Department of Cardiology Guy's and St Thomas' NHS Foundation Trust London UK

**Keywords:** body surface mapping, electrocardiographic imaging, ischemic cardiomyopathy, repolarization time, ventricular arrhythmia

## Abstract

**Background:**

The leading cause of death in patients with ischemic cardiomyopathy is sudden cardiac death caused by ventricular arrhythmias. Accurate determination of arrhythmic risk in these patients is vital to allow clinicians to take appropriate preventive measures.

**Objective:**

To review and summarize the literature on electrocardiographic imaging (ECGi) metrics that could be used to predict arrhythmic risk in patients with ischemic cardiomyopathy.

**Methods:**

A comprehensive literature search was performed to retrieve research articles on non‐invasive electrocardiographic mapping techniques. Inclusion criteria of the studies required the involvement of patients with ischemic cardiomyopathy or ischemic heart disease.

**Results:**

A total of 17 papers were identified, five of which specifically utilized ECGi to acquire metrics associated with an increased risk of ventricular arrhythmia (VA). ECGi metrics, including activation time, repolarization time, activation‐recovery interval, and voltage amplitude, were distinguishable between patients with ischemic cardiomyopathy, patients with a history of VA, and healthy controls.

**Conclusion:**

ECGi allows non‐invasive measurement of metrics which are associated with an increased risk of ventricular arrhythmias in patients with ischemic cardiomyopathy. ECGi may be a useful tool for risk assessment in these patients. Prospective studies are warranted for further validation and prediction of clinical endpoints.

## Graphical abstract

ECGi‐specific studies for assessment of arrhythmic risk in ischemic heart disease. Studies specifically using ECGi have identified several metrics that may be associated with ventricular arrhythmia risk in patients with ischemic cardiomyopathy. ARI, activation‐recovery interval; AT, activation time; ICM, ischemic cardiomyopathy; LV, left ventricle; MI, myocardial infarction; VT, ventricular tachycardia.

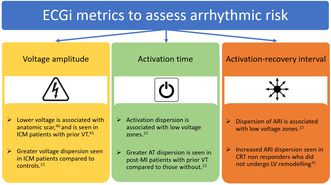

## 1 INTRODUCTION

Ischemic cardiomyopathy (ICM) is a weakening of myocardial contractility which is secondary to coronary artery disease. ICM is the leading cause of heart failure and continues to be associated with high morbidity and mortality despite contemporary medical and device therapy.^1^ The leading cause of death in this population is sudden cardiac death.^2^, ^3^

**FIGURE 1:**
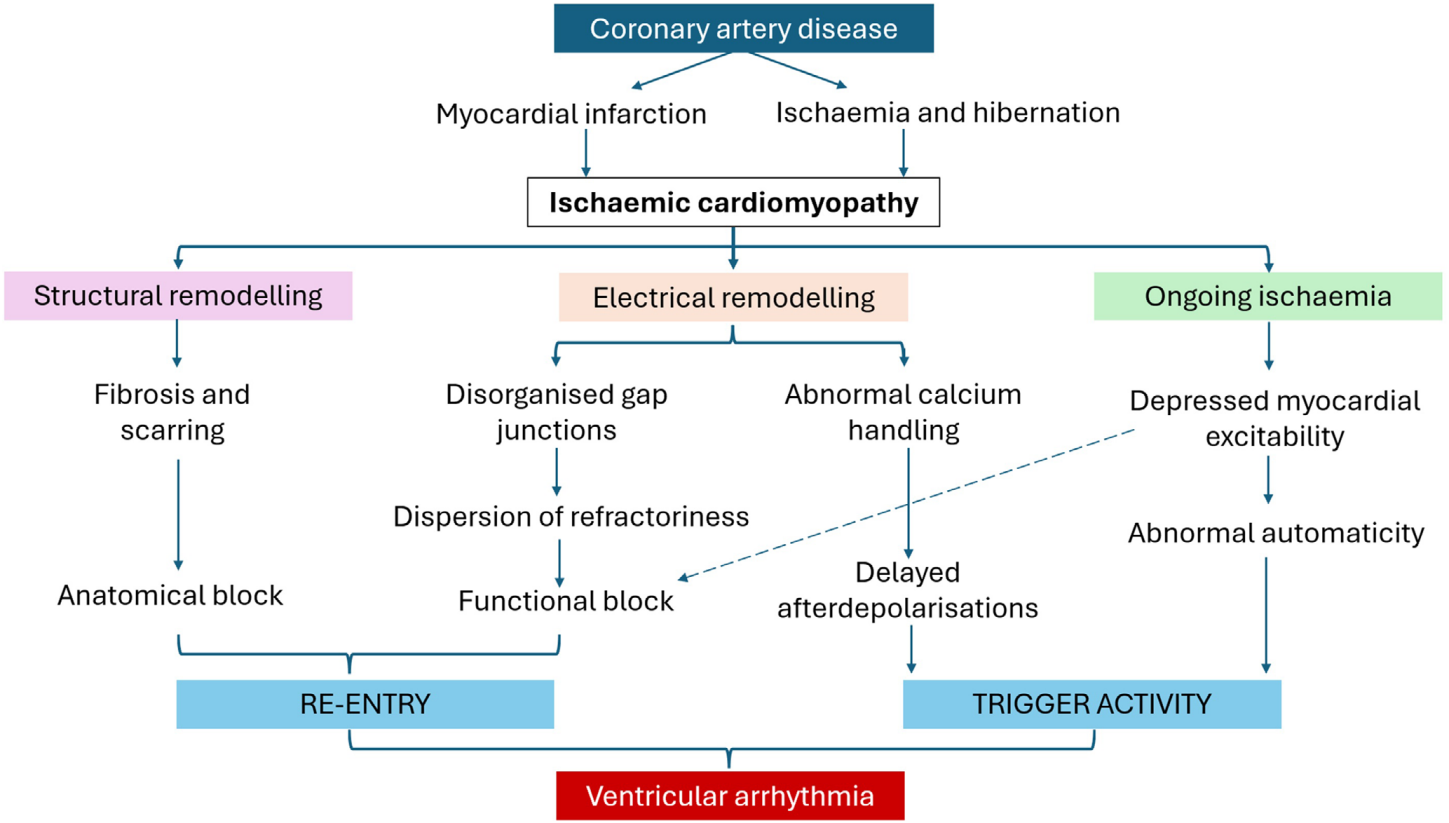
Pathophysiology of ventricular arrhythmia in ICM. Ventricular arrhythmogenesis requires substrates for both re‐entry and trigger activity. A re‐entry circuit can be formed due to anatomical block via structural remodeling as well as functional block due to electrical remodeling. Electrical remodeling also contributes to the formation of arrhythmic triggers.^4^, ^5^, ^6^, ^7^

Sudden cardiac death (SCD) in ICM is caused by fatal ventricular arrhythmias including ventricular tachycardia (VT) and ventricular fibrillation (VF). The mechanism of ventricular arrhythmia generation is related to an anatomical substrate and trigger activity created by ischemic changes (Figure 1).^4^, ^5^ First, the substrate, where replacement of functional cardiomyocytes with fibrosis blocks or delays the conduction of impulses through the myocardium and facilitates re‐entry.^4^ Second, the occurrence of a trigger, where an afterdepolarization initiates a re‐entry circuit.^6^, ^8^

Insertion of an implantable cardioverter defibrillator (ICD) is undertaken for primary and secondary prevention of SCD in patients with ICM. However, ICDs are associated with considerable cost as well as patient burden.^9^ During and after ICD insertion, complications such as infection, hematoma, pneumothorax, and inappropriate therapies can also occur.^10^, ^11^ It is therefore vital that clinicians are able to accurately assess SCD risk, ensuring those at risk are not left unprotected and conversely those unlikely to suffer fatal arrhythmias are not unnessarily exposed to an ICD.

Current guidelines stratify patients' SCD risk based on symptom burden, echocardiographic and ECG features. The latest European Society of Cardiology guidelines recommend ICD therapy in patients with ischemic heart disease and a left ventricular ejection fraction (LVEF) ≤35% despite 3 months of optimal medical therapy.^9^ This recommendation is derived from the MADIT and SCD‐HeFT trials that identified a significant mortality reduction in patients with reduced LVEF after they underwent ICD implantation.^12^, ^13^ However, since these historic trials, it is now evident that LVEF is an ineffective tool for SCD risk stratification with low levels of therapies observed in those receiving primary prevention devices.^14^ Furthermore, a significant incidence of SCD is reported in patients with LVEF >35% who were thus not eligible for ICD.^15^ There is therefore an urgent need to identify the improved predictors of arrhythmic risk.

Contemporary guidelines have repopularized the use of the electrophysiologic study (EPS) to aid risk assessment in those considered to be ‘borderline’ (LVEF 35–40%), advising ICD implantation only in those with inducible VT.^16^ While an EPS may be the gold standard to assess risk of ventricular arrhythmia, it is an invasive test associated with procedural risk as well as fiscal burden on the health service. Therefore, the development of a noninvasive, lower cost risk assessment alternative is very attractive.

Electrocardiographic imaging (ECGi) may provide an alternative solution to improve risk stratification. ECGi is a noninvasive method that acquires epicardial electrical activity from multiple leads (50–300) that are placed across the torso. Combined with geometric data from computed tomography or magnetic resonance imaging, cardiac maps of epicardial activation and repolarization can be generated.^17^ ECGi electrograms also provide quantitative metrics including activation time (AT), repolarization time (RT), activation‐recovery interval (ARI), and signal amplitude.

**FIGURE 2:**
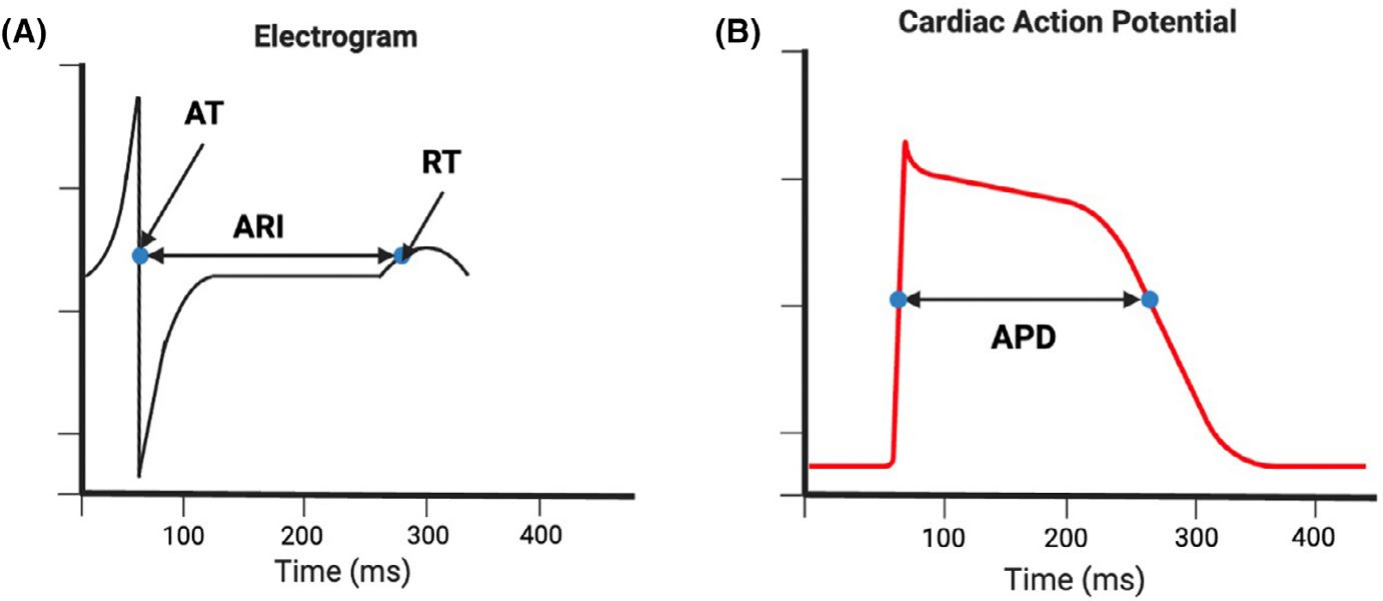
Derivation of metrics from an electrogram. (A) The activation time, repolarization time and activation‐recovery interval are measured from the unipolar electrograms acquired using ECGi. (B) The ARI measures the distance between activation and repolarization and is considered a surrogate for action potential duration.^18^

Calculation of AT and RT is performed using the Wyatt method, where the AT is the steepest downslope of the QRS complex and the RT is the maximum upslope of the T wave (Figure 2). The ARI can be determined by measuring the distance between AT and RT and is considered a surrogate of action potential duration (APD), which has been found to be higher within areas of myocardial scar and linked to arrhythmic risk.^19^ The dispersion of ARI in neighboring cardiomyocytes creates a functional block that can lead to re‐entrant arrhythmia^20^; this can be measured using the standard deviation of ARI measurements across unipolar electrograms.

Dispersion of RT and ARI have been found to be associated with a history of SCD and ventricular arrhythmia in patients with inherited arrhythmia syndromes.^21^, ^22^ Determining specific ECGi metrics that are associated with arrhythmogenesis would allow the stratification of patients at risk of ventricular arrhythmia and sudden cardiac death, identifying those most likely to benefit from ICD therapy without the need for an invasive procedure.

The aim of this review is to describe ECGi metrics that have been found to be associated with an increased risk of ventricular arrhythmia and SCD in patients with ischemic heart disease.

## 2 METHOD

A scoping review was undertaken to enable mapping of the literature related to this area. The search was conducted on PubMed on May 18, 2023; the full search strategy is detailed in the Table S1.

Papers were included if they met the following criteria: *Population*: human subjects with ischemic heart disease (IHD) with or without control populations; *Intervention*: acquisition of quantitative data from unipolar electrograms, *Design*: all clinical studies (excluding case reports, editorials or reviews), *Outcome*: arrhythmia risk or prior occurrence. Only full‐text papers published in English were included. The literature search was not limited to a specific publication time period to allow maximum acquirement of studies. Screening was conducted independently by two authors using Rayyan software. Any conflicts were resolved by discussion.

## 3 RESULTS

**FIGURE 3:**
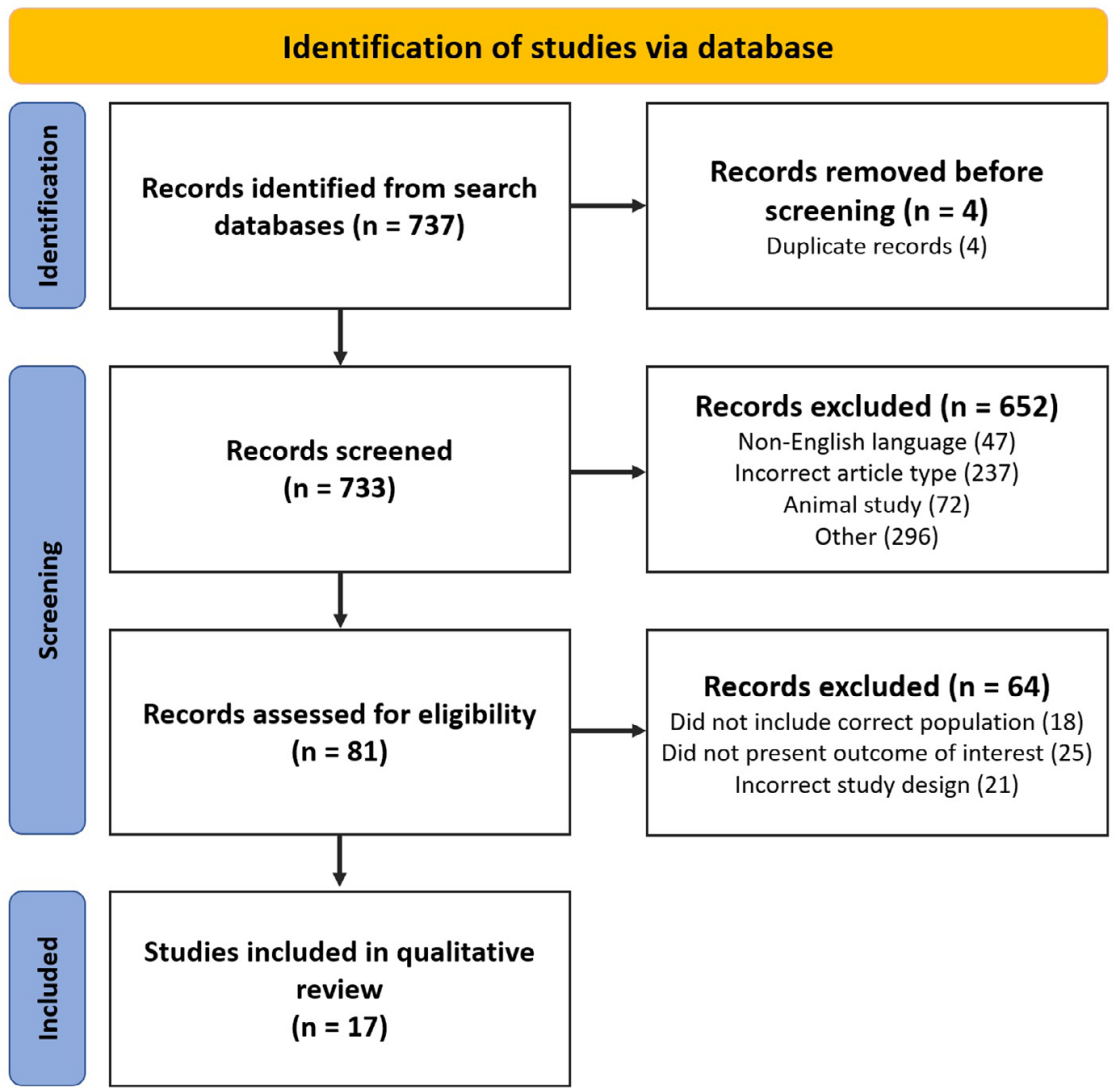
PRISMA flowchart for article screening. Article screening was performed according to PRISMA guidelines using Rayyan software.

In total, 737 studies were identified from the initial search (Figure 3). After title, abstract and full‐text review, 17 studies from 1995 to 2023 were selected, which included a total of 861 participants.

Five studies were identified that specifically used ECGi to obtain quantitative metrics (Table 1). Due to limited quantitative ECGi research, studies acquiring similar metrics but using multi‐lead surface ECGs, including body surface potential mapping (BSPM) and electroanatomic mapping (EAM) were also included (Table S2).

**TABLE 1:** ECGi‐specific studies.

Author	Population	Methods	ECGi metrics	Findings
Perez‐Alday et al. (2020, USA)^23^	10 ICM patients with VT, 10 ICM without VT, 10 HCM, 10 controls (88% white)	ECGi (128 electrodes)	Unipolar voltage, ventricular activation dispersion, voltage dispersion	Greater voltage dispersion seen in both ICM groups compared to healthy controls Local AT dispersion greater in VT group vs. non‐VT groups
Zhang et al. (2016, USA)^24^	17 ICM patients with VT, 15 ICM without VT	ECGi (256 electrodes)	EGM magnitudes, fractionation, presence of late potentials	VT group had lower normalized EGM magnitude, higher scar burden, greater prevalence of fractionated EGMs and late potentials
Cuculich et al. (2011, USA)^25^	24 patients with history of MI (87.5% white, 8.3% black, 4.2% other)	ECGi compared with scar in DE‐MRI and SPECT	EGM magnitude (voltage), EDM (electrogram deflection map)	55% of EGMs were low voltage, 19% very low voltage Epicardial electrical scar accurately detected anatomical scar
Elliott et al. (2022, UK)^26^	11 HF patients (7 ICM), LVEF ≤35%	ECGi (252 electrodes) Cardio‐Insight	Activation maps RT ARI cARI	LV cARI dispersion was higher 6 months post CRT compared to baseline in non‐responders. Qualitative maps confirmed increased heterogeneity
Graham et al. (2022, UK)^27^	16 patients with structural heart disease (7 IHD)	ECGi (252 electrodes) compared with EAM (CARTO)	Signal fractionation Activation dispersion (H_AT_) Repolarization Dispersion (H_ARI_)	Local dispersion of repolarization, ARI and AT were associated with bipolar voltages in EAM. Signal fractionation, H_AT_ and H_ARI_ were associated with invasive low‐voltage zones

Abbreviations: ARI, activation‐recovery interval; AT, activation time; cARI, corrected ARI; CRT, cardiac resynchronization therapy; DE‐MRI, delayed‐enhanced magnetic resonance imaging; EAM, electroanatomic mapping; EGM, electrograms; EP, electrophysiology; HCM, hypertrophic cardiomyopathy; HF, heart failure; ICM, ischemic cardiomyopathy; IHD, ischemic heart disease; LVEF, left ventricular ejection fraction; MI, myocardial infarction; RT, repolarization time; SPECT, single‐photon emission computerized tomography; VT, ventricular tachycardia.

### 3.1 Individual metrics

#### 3.1.1 Activation time

Multiple studies assessed activation time and patterns using ECGi. Perez‐Alday et al. compared patients with and without a history of VT, identifying no significant differences in AT but increased AT dispersion in patients with VT. The authors concluded this likely indicated the presence of scar which led to more varied conduction patterns.^23^ These findings were supported by Graham et al., who found that AT heterogeneity was negatively correlated with bipolar EAM voltage, which is known to be strongly correlated with scar. This study also reported an association between low voltage zones with the combination of signal fractionation and heterogeneity of ARI and AT.^27^

Previous research using related non‐invasive modalities, comprising of BSPM, signal averaged‐ECG (SAECG) and magnetocardiography (MCG), studied post‐MI patients with and without VT and identified longer QRS durations, increased root mean square of the QRS (RMS), and an increased proportion of low signal amplitudes in those had experienced VT.^28^

#### 3.1.2 Repolarization time

Invasive work has previously identified increased RT and RT dispersion in scar regions compared to normal tissue in patients with ICM.^18^ Early studies of multiple body surface ECGs attempted to study dispersion of the QT interval as a surrogate of repolarization heterogeneity.^29^ Stellbrink et al. studied repolarization in patients with coronary artery disease (CAD) using both 12‐lead ECGs and 62‐lead BSPM.^30^ While no difference in QT interval dispersion was seen on the 12‐lead ECG, the corrected QT (QTc) dispersion was longer in CAD patients on BSPM. However, no difference was seen between CAD patients with and without a history of arrhythmia.^30^ In contrast, Nirei et al. measured RT dispersion in post‐MI patients with and without history of VT and identified higher corrected repolarization time (RTc) gradients in the VT positive cohort, driven by a lower minimum RTc.^31^ Shorter RTs have also been reported in patients with a high burden of PVCs^32^ and in survivors of idiopathic VF, with larger areas of early repolarization increasing vulnerability to VF.^33^, ^34^

Another metric to assess both activation and repolarization heterogeneity is the QRST gradient. Goldner et al. identified lower mean QRST gradients in the VT cohort as well as greater low‐amplitude signal duration;^35^ however, similar work assessing QRST‐area and QRST integral maps failed to find significant differences in mean values between groups.^36^, ^37^

Fereniec et al. proposed new parameters to assess arrhythmic risk in post‐MI patients, including STT QRS correlation, STT dispersion and TSI dispersion and reported a lower mean of STT QRS correlation and higher STT and TSI dispersion in those who had VT compared to those who had not.^38^ Reported RT assessments using ECGi are limited; Elliot et al. did not identify significant alterations in RT or RT dispersion in response to CRT,^26^ while Graham et al. identified a correlation between RT dispersion and signal amplitude.^27^

#### 3.1.3 Activation‐recovery interval

Increased ARI dispersion is a popular metric as it has been linked to the formation of re‐entrant arrhythmias.^20^ On BSPM, sympathetic activation has been found to shorten ARI, QT interval and T‐wave amplitude in patients with and without structural heart disease.^39^ Using ECGi, Elliot et al. studied the effect of LV reverse remodeling on ARI metrics,^26^ identifying increased ARI dispersion at 6 months in patients who had not responded to CRT; with LV remodeling an independent predictor of increased LV ARI dispersion.^26^ Higher ARI variability was also seen in ICM patients who had documented ventricular arrhythmias on CRT‐D compared to those free from arrhythmia; this was seen independently of LVEF which was not correlated to ARI dispersion.^40^ Invasive assessments of patients with scar‐related VT, using both endocardial and epicardial mapping, has suggested that ARI prolongation should be assessed according to regions of myocardial scar as opposed to global metrics, which may dilute subtle alterations.^19^

Invasive work using contact mapping by Callans et al. reported shorter endocardial ARI in VT re‐entry circuits compared to areas outside the circuit despite being in the same region of scar.^41^ Other work has found that endocardial measurements of ARI and ARI dispersion using CARTO did not show significant difference between patients with high burden of PVC and controls.^32^ However, in the same study, ECGi detected lower minimum ARI and greater ARI dispersion in the high burden PVC group without structural heart disease.^32^

#### 3.1.4 Other metrics

Earlier studies using BSPM identified larger areas of low amplitude signal in patients with spontaneous or inducible sustained VT compared to those without sustained VT.^35^ Wang et al. conducted invasive and ECGi assessment in a cohort of patients with scar‐related VT and found ECGi was able to correctly identify areas of low voltage and signal fractionation.^42^

This was supported by work using MRI and SPECT imaging, which found that ECGi could accurately detect the presence of scar with a high sensitivity (89%) and specificity (85%).^25^ They identified the importance of individualized metrics, reporting that signal amplitudes lower than 30% of that's individual's highest amplitude should be considered scar, as opposed to a standard value for all patients.^25^ Other authors have measured signal fractionation on ECGi, identifying a higher proportion of total and dense scar in VT positive cohorts, with a higher percentage of fractionated EGMs within scar and lower unipolar voltages compared to patients without VT, this was independent of LVEF.^24^ This is supported by invasive work that reported fractionated electrograms and lower voltages in ICM patients with inducible VT.^43^ Variation in voltage dispersion was also seen on ECGi between healthy controls, post‐MI patients with and without VT and patients with hypertrophic cardiomyopathy, with the largest values seen in the latter.^23^

## 4 DISCUSSION

In this review, a small sample of studies utilizing ECGi to non‐invasively assess arrhythmic risk in patients with ICM have been identified. The ECGI metrics identified to be associated with ischemic cardiomyopathy and arrhythmic risk are prolonged AT, greater AT dispersion, mean ARI and ARI dispersion and lower voltage or signal amplitude (Table S3).^23^, ^24^, ^25^, ^26^, ^27^, ^40^

Prolonged AT and greater AT dispersion assessed by EP study has also been reported to be associated with SCD and sustained VT in patients with idiopathic dilated cardiomyopathy patients.^44^ AT dispersion may be a useful marker of arrhythmic risk in ICM patients; porcine models of myocardial infarction have suggested that prolongation of AT in affected areas immediately after the occlusion of the left anterior descending coronary artery is associated with the incidence of VF.^45^ The difference in AT between healthy and ischemic tissues creates dispersion which then promotes arrhythmogenesis, and this may explain why reduction of dyssynchrony with CRT has been shown to reduce death in ICM populations.^46^

Alteration in repolarization logically predisposes to arrhythmogenesis by allowing the formation of a re‐entry circuit. It is therefore most appealing to focus on metrics of repolarization when considered arrhythmic risk. However, as shown here, studies to date have gleaned mixed results.^30^, ^31^ It is possible that the technology or the metric may not be specific enough to distinguish the risk of arrhythmia within higher risk groups.

Invasive work previously identified both mean ARI and ARI dispersion to be altered in patients with ischemic heart disease.^41^ Studies identified here have not detected differences in mean ARI; however, ARI variability was associated with arrhythmic risk, scar burden and LV remodelling.^26^, ^40^ This is supported by work in patients with VF but structural normal hearts.^21^ It may be that ECGi lacks the spatial resolution to locate ARI variation, or it may be a limitation of global metrics. Within post‐infarction areas that have undergone electrical remodeling, changes in ion channel availability and dysfunctional gap junctions can shorten the action potential duration and therefore the ARI. Therefore, mean ARI may be better assessed locally in regions of myocardial scar rather than globally, as healthy regions could disrupt mean values.^19^

Scar burden is associated with an increased risk of ventricular arrhythmia and SCD in patients with ICM.^47^, ^48^ As ECGi can also determine scar burden, it could also be used for risk stratification in geographical areas where access to advanced imaging is challenging. However, further studies assessing a direct correlation between ECGi‐detected scar burden and incidence of ventricular arrhythmia should be conducted.

Our review has some limitations. The majority of studies included had a small sample and therefore did not have high statistical power to determine significance. Unequal sample sizes were also found in several studies which may have further affected power.^32^, ^37^, ^38^ We acknowledge that the study populations were predominantly composed of white individuals, which limits the generalizability of the findings. This is particularly relevant as ethnicity may influence the risk factors for ventricular arrhythmia and sudden cardiac death. Although it was appealing to conduct a meta‐analysis of the quantitative metrics, the number of studies identified were small, and metrics reported were varied, including whether these had or had not been corrected for heart rate. The large number of records excluded may rise concern of selection bias; however, the majority were excluded during initial screening as they did include the population, method, or outcome of interest.

### 4.1 Future work

Re‐entry vulnerability index (RVI) represents another possible metric of interest, as it measures the risk of re‐entry due to alteration in activation and repolarization of cardiomyocytes. It is determined by subtracting AT from RT and can determine the presence of functional substrates of arrythmia. A shorter repolarization relative to activation will allow an impulse to re‐enter the circuit, and thus, low RVI represents higher risk of re‐entry and arrhythmia.^49^ A study by Orini et al. aimed to study the correlation of RVI with VT sites of origin by using electroanatomic mapping before VT ablation procedures; they identified that regions of low RVI were clustered around the site of VT origin. Furthermore, RVI had higher accuracy to determine VT sites of origin as compared to maximum ARI gradients.^50^ ECGi acquires both AT and RT and so could be utilized for RVI measurement; however, there are limited data regarding its use to derive this metric, and whether it would add any additional information above ARI and ARI dispersion.

### 4.2 Clinical relevance

ECGi metrics have been identified that are associated with the occurrence of ventricular arrhythmias, including greater dispersion in voltage amplitude and ARI repolarisation.^23^, ^24^, ^25^, ^26^, ^27^ In clinical settings, patients being considered for an ICD could undergo ECGi and have these metrics assessed to aid in arrhythmic risk stratification. However, prior to clinical use of ECGi, further validation including prospective studies with clinical endpoints will be required. A standardized operating procedure as well as normal ranges for each metric would also be required in order to ensure uniformity and accurate identification of patients with higher arrhythmic risk. Key metrics that are found to predict the occurrence of ventricular arrhythmias and SCD could then be used to stratify patients for ICD insertion. This would be particularly relevant in healthcare settings where the cost or availability of methods such as cardiac MRI and EPS limit their use.

## 5 CONCLUSIONS

ECGi‐derived metrics including activation dispersion, ARI dispersion, voltage dispersion, prevalence of fractionated signals, and lower signal amplitude have all been identified to be associated with arrhythmic risk in patients with ischemic heart disease. Whether these metrics are able to prospectively predict risk of arrhythmia remains to be determined.

## FUNDING INFORMATION

This analysis was supported by the British Heart Foundation (Fellowship FS/CRTF/21/24190 and the King's BHF Center of Research Excellence grant RE/18/2/34213).

## CONFLICT OF INTEREST STATEMENT

Authors declare no conflict of interests for this article.

## ETHICS STATEMENT

None.

## PATIENT CONSENT STATEMENT

None.

## CLINICAL TRIAL REGISTRATION

None.

## PERMISSION TO REPRODUCE MATERIAL FROM OTHER SOURCES

None.

## Supporting information


Data S1:

## Data Availability

None.
