# Supplementary material for: Electrocardiographic imaging metrics to predict the risk of arrhythmia in patients with ischemic cardiomyopathy
Source: J Arrhythm. 2025 Feb 17;41(1):e70024. doi: 10.1002/joa3.70024 (PMC11831206; doi:10.1002/joa3.70024)
Supplement: Supplementary file 1 — Data S1: [file JOA3-41-e70024-s001.pdf]

Supplementary Material

Table S1. Search strategy

**Search strategy**	(“Ventricular Arrhythmia”[All Fields] OR “Tachycardia, Ventricular”[MeSH] OR “Ventricular Fibrillation”[MeSH]) AND (“ECGi”[All Fields] OR “Electrocardiographic Imaging”[All Fields] OR “Electrocardiographic Mapping”[All Fields] OR “Body Surface Potential Mapping”[MeSH]) AND (“Ischemic cardiomyopathy”[All Fields] OR “Ischemic”[All Fields] OR “Ischemia”[All Fields] OR “Myocardial Infarction”[MeSH] OR “Myocardial Ischemia”[MeSH])
**Study types**	- Clinical study<br>- Clinical trial<br>- Comparative study<br>- Controlled clinical trial<br>- Observational study<br>- Randomized controlled trial

Table S2. Supporting studies

Author	Population	Method	Metrics	Findings
**Cluitmans et al. (2021, Netherlands)[29]**	Ex-vivo (6 porcine, 1 human)<br>Human (11 VF patients)	BSPM<br>184 lead (BioSemi)	AT<br>RT<br>RTg	Repolarization substrate, defined as RTG >200ms/cm were found significantly more frequently in idiopathic VF group (0.02)
**Parreira et al. (2019, Portugal)[28]**	7 patients with frequent PVC<br>17 controls	12 lead ECG,<br>224 electrodes (Amycard 01C EP),<br>ECG-gated CT scan	AT<br>RT<br>ARI	Patients with PVC had shorter median RT and median minimal ARI. They also had longer ΔARI and ΔRT compared to control.<br>ARI was linearly associated with RT.
**Wang et al. (2018, USA)[39]**	4 patients (1 excluded due to excessive noise)	120 lead ECG<br>CT Scan	Voltages (<0.5mV – scar)	ECGi identified areas of signal fractionation and low voltage, correlated with invasive findings, and allowed mapping of reentry circuits.
**Fereniec et al. (2011, Poland)[34]**	26 MI patients with VT<br>14 MI patients without VT<br>25 controls	64-leads ECG (Active two, BioSemi)	STT_QRST_CORR,<br>STT_DI<br>TSI_DI	Statistically significant lower mean of STT_QRST_CORR in MI VT patients compared to non-VT.<br>Significantly higher STT_DI, TSI_DI, QRS interval, QT interval in MI with VT compared to without VT.
**Kozmann et al. (2010, Hungary)[30]**	6 VA patients with ICD<br>14 controls	BSPM (64 electrodes) BioSemi	Beat to beat QRS<br>QRST integral maps	Plotting of QRST integral map identified large differences in repolarisation depicted by dispersed distribution of dipolar components in arrhythmic patients compared to healthy subjects.
**Korhonen et al. (2002, Finland)[23]**	44 patients with LVEF≤45% and history of MI<br>(22 patients had VT)	MCG,<br>BSPM 63 leads	QRS duration<br>RMS<br>LAS	Late fields in MCG and late potentials in both BSPM and SA-ECG identify patients with higher risk of arrhythmia.<br>LAS in the VT group was significantly greater than control in MCG, BSPM, and SAECG.
**Nirei et al. (2001, Japan)[27]**	40 patients with anterior MI (20 patients had VT)	BSM (87 electrodes)	RT isochrone map	RTc dispersion was smaller in MI patients without sustained VT. Max RTc among those 2 groups did not differ but min. RTc was significantly

	40 DCM patients (20 with VT)<br>40 control		RTc dispersion	lower in MI with VT. Overall - the dispersion was greater in MI with VT compared to without VT.
**Shusterman et al. (1999, USA)[36]**	SHD group: 18 patients (6 had CAD and 2 had CHF)<br>Non SHD: 36 patients	BSPM (32 electrodes/192 points SAECG)	QT interval<br>ARI TWA<br>QRST-area integral<br>T-wave area integral	ARI shortened in both groups significantly after sympathetic activation, TWA increased. But TWA was not altered in SHD group. This suggested that structural abnormalities disturb sympathetic receptors.
**Stroink et al. (1999, Canada)[33]**	76 controls<br>15 MI patients<br>15 VA patients (12 had IHD)	Magnetic field map<br>BSPM	QRST integral maps	No differences between the QRST integral maps of MI and VA group. Though both of those groups had significantly larger nondipolar content compared to controls.
**Stellbrink et al. (1999, Germany)[26]**	I: 22 CAD+VF<br>II: 21 CAD no VF<br>III: 13 idiopathic VF<br>IV: 18 healthy controls	BSPM (62 electrodes) compared to 12-lead ECG	QRST integral maps<br>QT dispersion	No difference in QT dispersion and interval between groups on ECG. With BSPM, QTc dispersion was greater in idiopathic VF and QT interval was longer in CAD patients with or without VF compared to healthy control.
**Goldner et al. (1999, USA)[31]**	Group 1=26 patients with NSVT<br>Group 2=27 patients with spontaneous or inducible sustained VT	BSPM (32 electrodes/192 points SAECG)	QRST isoarea gradient	QRST iso area gradient range was lower in group 2. Mean QRST duration and low amplitude signal duration was longer in group 2. Univariate predictors of VT →LVEF ≤40%, CAD, age ≥60 years, QRST isoarea gradient range ≤109mv/ms and SAECG filtered QRS duration >114 ms.
**Hubley-Kozey et al. (1995, Canada)[32]**	102 VT group<br>102 MI non-VT	120 leads ECG, BSPM, 3 limb leads, 117 unipolar chest leads	QRS duration<br>QTc	Subjects in VT group had poorer LV function, longer QRS duration and QTc interval, higher HR during sinus rhythm than non-VT group.

ARI= activation-recovery interval, AT= activation time, BSPM= body surface potential mapping, CAD= coronary artery disease, CHF= chronic heart failure, DCM= dilated cardiomyopathy, ECG = electrocardiography, ECGi= electrocardiographic imaging, EGM= electrograms, HR= heart rate, IHD= ischemic heart disease, LAS= low amplitude signal, LVEF= left ventricular ejection fraction, MCG= magnetocardiography, MI= myocardial infarction, NSVT= non-sustained

ventricular tachycardia, PVC= premature ventricular complexes, RT= repolarisation time, RTg= RT gradient, SAECG= signal averaged electrocardiography, SHD= structural heart disease, TWA= T-wave amplitude, VA= ventricular arrhythmia, VT= ventricular tachycardia.

Table S3. Key findings by ECGi metric

	No. of patients	Pathology	Primary outcome	Key finding
Activation dispersion				
**Graham et al. (2022, UK)[22]**	16	Structural heart disease (50% IHD)	AT dispersion – correlation with invasive mapping	Effect size -0.003 (95% CI -0.005 to -0.002), p=<0.05
**Repolarisation** dispersion				
**Parreira et al. (2019, Portugal)[28]**	24	Frequent PVCs vs control	RT dispersion (Δ RT)	Δ RT : 201 (160-235) vs 115 (65-177), p=0.019
ARI / ARI dispersion				
**Parreira et al. (2019, Portugal)[28]**	24	Frequent PVCs vs control	ARI dispersion (Δ ARI)	Δ ARI : 145 (68-216) vs 17 (3-48) ms, p = 0.001
**Hubley-Kozey et al. (1995, Canada)[32]**	204	VT (76% IHD) vs prev MI but no VT	QRST area	Sensitivity 90% and specificity 78% for VT
**Fereniec et al. (2011, Poland)[34]**	65	MI with VT vs MI without VT (vs controls)	STT QRS correlation (STT QRS C)	STT QRS C : 0.35 v 0.63, p=0.05 (vs 0.91, p=0.004)
**Elliott et al. (2022, UK)[35]**	11	HF patients (63.6% ICM) – CRT responders v non responders	LV ARI dispersion (Δ LV ARI) baseline vs 6 months post CRT	Δ LV ARI : 22.6 ± 2.6 ms vs 38.3 ± 1.2 ms; p = 0.004
**Voltage size/signal amplitude**				
**Perez-Alday et al (2020, USA)[21]**	40	HCM vs ICM- VT vs ICM-no VT v controls	Voltage dispersion (Δ Voltage)	Δ Voltage : 215 vs 189 vs 158 vs 110, p=0.041
**Cuculich et al (2011, USA)[40]**	24	IHD, mean LVEF 30%	Reduced signal amplitude (low voltage)	89% sensitivity and 85% specificity at detecting scar compared to SPECT
**Zhang et al (2016, USA)[41]**	32	ICM-VT v ICM-noVT	EGM magnitude/voltage (EGM v)	EGM v : 0.107 ± 0.027 vs 0.153 ± 0.031, p < 0.05

ARI= activation-recovery interval, AT= activation time, CI = confidence interval, CRT= cardiac resynchronization therapy, HCM= hypertrophic cardiomyopathy, HF= heart failure, ICM= ischemic cardiomyopathy, IHD= ischemic heart disease, LV= left ventricle, MI= myocardial infarction, PVC = premature ventricular contraction, RT = repolarisation time, VT= ventricular tachycardia
